# Redox Imbalance and Genetic Mutations in Heart Failure: Synergistic Mechanisms and Therapeutic Strategies

**DOI:** 10.3390/genes17020225

**Published:** 2026-02-11

**Authors:** Vinod Kumar Balakrishnan, Abinayaa Rajkumar, Monisha K. G. Ganesh, Harilalith Reddy Kovvuri, Durgadevi Selvam, Preetam Krishnamurthy, Sandhya Sundaram, Kalaiselvi Periandavan, Sankaran Ramesh, Muralidharan Thoddi Ramamurthy, Namakkal S. Rajasekaran

**Affiliations:** 1 Department of Cardiology, Sri Ramachandra Institute of Higher Education and Research, Chennai 600116, Tamil Nadu, India; 2 Department of Medical Biochemistry, Taramani Campus, University of Madras, Chennai 600113, Tamil Nadu, India; 3 Department of Cardiovascular Technology, Faculty of Allied Health Sciences, Sri Ramachandra Institute of Higher Education and Research, Chennai 600116, Tamil Nadu, India; 4 Department of Pathology, University of Alabama at Birmingham, Birmingham, AL 35233, USA; 5 Department of Pathology, Sri Ramachandra Institute of Higher Education and Research, Chennai 600116, Tamil Nadu, India; 6 Institute of Cardiac Sciences, Department of Cardiology, SRM Medical College, Kattankulathur, Chengalpattu 603203, Tamil Nadu, India; 7 Division of Molecular and Cellular Pathology, Department of Pathology, University of Alabama at Birmingham, Birmingham, AL 35233, USA

**Keywords:** heart failure, redox imbalance, oxidative stress, genetic mutations, cardiomyopathy, mitochondrial dysfunction, redox signaling

## Abstract

Heart failure (HF) is a significant global health challenge, with rising prevalence and a complex, multifactorial pathophysiology. Emerging evidence suggests that disruptions in redox signaling pathways and genetic mutations play critical, synergistic roles in the development and progression of HF. This comprehensive review synthesizes current knowledge on how redox imbalance and genetic alterations interact to drive cardiac dysfunction and critically evaluates the therapeutic strategies targeting these mechanisms. We begin by introducing the basic concepts of redox biology and its role in maintaining cardiac homeostasis. Next, we examine the specific redox signaling pathways and genetic mutations implicated in HF pathogenesis, highlighting the latest mechanistic insights and findings from human studies. The complex interplay between redox dysregulation and genetic factors is explored, including their synergistic effects, compensatory mechanisms, and illustrative case studies. We also review current therapeutic strategies aimed at restoring redox balance and correcting underlying genetic mutations, discussing their progress and limitations. Finally, we present the latest research advances, identify critical knowledge gaps, and propose future directions for both basic and translational research. Understanding the redox–genetic axis is key to developing novel, targeted therapies to address the growing HF epidemic.

## 1. Introduction

Heart failure (HF) is a progressive clinical syndrome characterized by the heart’s inability to pump sufficient blood to meet the body’s metabolic demands. HF affects approximately 6.2 million adults in the U.S., projected to exceed 8 million by 2030 [1]. Despite therapeutic advances, the prognosis for HF remains poor, with 5-year survival rates under 50% [2]. The pathophysiology of HF is multifactorial, involving genetic, metabolic, structural, and environmental factors that impair cardiac function. Understanding the molecular mechanisms driving HF is critical for developing targeted therapies to improve patient outcomes [3].

Among the many contributing factors, redox signaling has emerged as a central player in HF pathogenesis. This process involves the reversible modification of proteins and other macromolecules by reactive oxygen and nitrogen species (ROS/RNS) [4]. Physiological redox signaling is essential for normal cardiac function, regulating processes such as excitation–contraction coupling, metabolism, and cell survival [5]. However, excessive ROS/RNS production leads to chronic oxidative/nitrosative stress, a hallmark of failing hearts [6]. Genetic and epigenetic alterations within the myocardium also play a significant role in HF, with many mutations linked to cardiomyopathies and redox homeostasis [7,8].

Despite substantial research into both redox signaling and genetic contributions to HF, their precise interconnections remain incompletely understood. Most studies have focused on either redox or genetic mechanisms in isolation, limiting our understanding of their synergistic interactions. Advances in genomic and molecular technologies have provided new insights that have yet to be integrated into the existing framework of HF research. Thus, a comprehensive review of the redox–genetic interactions in HF pathogenesis is urgently needed.

This review aims to explore the dysregulation of redox signaling and genetic alterations in HF, evaluate their interplay, and discuss therapeutic opportunities and challenges. We introduce the basics of cardiac redox biology, outline the major redox signaling pathways and genetic mutations associated with HF, and highlight case-specific findings. Finally, we review current therapeutic approaches, recent advances, and future directions in HF research. Our goal is to provide a reference that the importance of the redox–genetic axis in HF pathogenesis, fostering new insights and therapeutic innovations to combat the global burden of HF [9].

To support this integrative overview, the present narrative review draws on the literature identified through targeted searches of PubMed, Scopus, and Web of Science, using combinations of keywords related to heart failure, redox signaling, oxidative and reductive stress, reactive oxygen species, genetic mutations, cardiomyopathy, and mitochondrial dysfunction. In addition, relevant studies were identified by manual examination of reference lists from key review articles and seminal original investigations. Study selection was guided by relevance to cardiac redox biology, genetic mechanisms, and translational implications in heart failure, with emphasis on peer-reviewed experimental, translational, and clinical studies that provide mechanistic insight or clinical relevance. As this article is intended as a narrative synthesis, no formal systematic review framework or quantitative meta-analysis was applied; instead, the literature was evaluated and discussed based on biological plausibility, consistency across studies, and contribution to understanding the redox–genetic axis in heart failure.

## 2. Redox Signaling in Heart Failure

### 2.1. Basic Redox Biology Concepts

Redox signaling primarily involves the reversible transfer of electrons between biomolecules, often leading to the modification of protein conformation and function. Key reactive oxygen species (ROS) include superoxide (O_2_^•−^), hydrogen peroxide (H_2_O_2_), and hydroxyl radicals (^•^OHs) [10], which are generated from mitochondria, NADPH oxidases (NOXs), xanthine oxidase, and uncoupled nitric oxide synthases (NOSs). Antioxidant defenses, such as superoxide dismutase, catalase, and glutathione peroxidase, neutralize ROS to maintain redox balance [11].

In physiological conditions, redox signaling regulates key processes like contractility, mitochondrial function, and cellular metabolism. However, when ROS production exceeds antioxidant capacity, oxidative stress occurs, leading to cellular damage. This imbalance significantly impacts cardiac function, particularly in the context of HF. The high mitochondrial content of the heart and reliance on redox-based metabolism make it especially sensitive to perturbations in redox homeostasis. Pathological redox signaling contributes to key aspects of the failing heart phenotype, including contractile dysfunction, calcium dysregulation, cardiomyocyte hypertrophy, apoptosis, arrhythmia, fibrosis, and chamber dilation. Additionally, ROS are involved in ischemia-reperfusion injury which is a major precursor for heart failure as well as in protective signaling pathways activated during stress. Moreover, it is known that the ROS levels and activity are increased in failing human heart and *Nox2* expression is also associated with activation of MAPK signaling pathways, further implicating redox imbalance in HF pathogenesis [11].

### 2.2. Major Redox-Sensitive Pathways Regulating Cardiac Function

Preclinical evidence, primarily from cellular and animal models, indicates that excessive reactive oxygen species disrupt multiple redox-sensitive proteins and signaling pathways essential for maintaining cardiac homeostasis, thereby contributing to structural and functional remodeling in heart failure. One major pathway affected is excitation–contraction coupling (ECC), the process by which electrical stimuli (action potentials) lead to myocardial contraction. According to Bers et al. [12], ECC begins with depolarization of the cardiomyocyte membrane, which opens L-type calcium channels and allows calcium influx. This influx triggers a much larger release of calcium from the sarcoplasmic reticulum via ryanodine receptor 2 (RyR2), a process known as calcium-induced calcium release. The resulting increase in cytosolic calcium binds to troponin C, enabling sarcomeric contraction.

In heart failure, oxidative stress modifies redox-sensitive cysteine residues on RyR2, increasing its open probability and leading to excessive calcium release. This results in intracellular calcium overload and impaired myocardial contractility [12]. In addition, reactive oxygen species interact with nitric oxide to form peroxynitrite, which enhances interleukin-6 receptor (IL6R) expression and subsequently downregulates transcription of the *ATP2A2* gene, reducing levels of **sarcoplasmic reticulum Ca^2+^-ATPase 2a (SERCA2a) [13]. Phospholamban (PLN), a key regulator of SERCA2a activity, is further influenced by NADPH oxidase 2 (NOX2) activation, elevated B-type natriuretic peptide (BNP), and post-translational modifications such as oxidation and O-GlcNAcylation [12,13]. Reduced ATP synthesis, commonly associated with mitochondrial dysfunction in heart failure, further compromises SERCA2a activity [13].

Oxidative stress also targets myofilament proteins, including troponin I and myosin-binding protein C, resulting in decreased force generation and myocardial contractility [14,15]. Within mitochondria, reactive oxygen species damage components of the electron transport chain (ETC), impair oxidative phosphorylation, reduce ATP production, and promote apoptotic signaling [16,17]. At the level of the extracellular matrix (ECM), oxidative stress activates matrix metalloproteinases (MMPs), facilitating matrix degradation, fibrosis, and pathological chamber dilation [18,19].

Furthermore, reactive oxygen species modulate intracellular kinases such as protein kinase A (PKA), protein kinase C (PKC), and mitogen-activated protein kinases (MAPKs), thereby driving maladaptive hypertrophy and apoptosis [20,21]. Oxidative stress also activates redox-sensitive transcription factors, including nuclear factor kappa B (NF-κB), activator protein-1 (AP-1), and nuclear factor erythroid 2–related factor 2 (Nrf2), which regulate genes involved in inflammation, fibrosis, and antioxidant defense, thereby perpetuating the heart failure phenotype [22,23].

**Figure 1:**
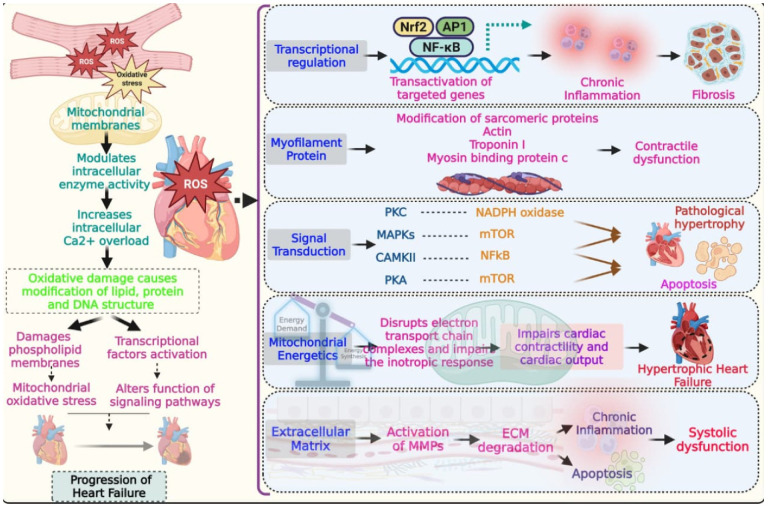
Redox–genetic mechanisms driving myocardial remodeling and heart failure.

These pathways are highly interconnected, and their dysregulation by redox imbalance leads to progressive cardiac dysfunction in heart failure. For instance, oxidative-stress-induced RyR2 calcium leak exacerbates sarcoplasmic reticulum calcium cycling defects, while convergent mitochondrial ROS signaling impairs energetics and promotes fibrosis (Figure 1). Mechanistically, mechanical stretching of cardiomyocytes activates plasma membrane NADPH oxidase, generating ROS that oxidize critical RyR2 cysteine residues and thereby enhance Ca^2+^-induced Ca^2+^ release, further amplifying this maladaptive Ca^2+^–ROS feedback loop [24]. Beyond this global dysregulation of Ca^2+^, accumulating evidence shows that TRP channels mediate local calcium signals, contributing to pathological hypertrophy and heart failure. Notably, TRPC3 has been reported to act as a positive regulator of NADPH oxidase 2, thereby amplifying ROS-dependent maladaptive signaling triggered by mechanical stretch during diastolic function in cardiomyocytes [25].

Excess reactive oxygen species disrupt mitochondrial function, calcium homeostasis, and intracellular signaling, leading to oxidative modification of lipids, proteins, and DNA. Activation of redox-sensitive transcription factors and post-translational modification of sarcomeric proteins impair contractility, while dysregulated signaling pathways promote hypertrophy, apoptosis, and extracellular matrix remodeling, collectively accelerating adverse cardiac remodeling and heart failure progression.

### 2.3. Oxidative Stress and Cardiac Dysfunction in Patients with HF

The findings discussed in this section are primarily derived from studies conducted in patients with heart failure, including analyses of human myocardial tissue, circulating biomarkers, and clinical observational cohorts, thereby providing direct clinical relevance to the mechanisms described. Numerous studies have documented increased systemic and myocardial oxidative stress markers in patients with heart failure [13]. Elevated plasma lipid peroxides and reduced antioxidant capacity correlate with disease severity and poorer clinical outcomes [14].

Myocardial levels of reactive oxygen species (ROS), including superoxide, are increases in failing hearts, and markers of oxidative damage such as nitrotyrosine and 4-hydroxynonenal (4-HNE) are associated with adverse myocardial remodeling [15,16,17,18,19]. Oxidative stress in heart failure is further amplified by neurohormonal and inflammatory stimuli. Activation of the renin–angiotensin–aldosterone system promotes ROS production through NADPH oxidase, while inflammatory cytokines such as tumor necrosis factor-α (TNF-α) exacerbate oxidative injury [20,21].

Experimental and translational studies indicate that oxidative stress plays a critical role in the progression from compensated hypertrophy to overt heart failure [22]. For example, pressure overload models demonstrate early increases in myocardial ROS, and attenuation of ROS generation can delay or prevent subsequent cardiac dysfunction. In clinical settings, oxidative stress is commonly assessed using biomarkers such as malondialdehyde (MDA), reduced glutathione (GSH) and oxidized glutathione (GSSG), as well as antioxidant enzyme activities including superoxide dismutase (SOD), glutathione peroxidase (GPx), and catalase (CAT) [26]. In cardiac tissue, thiol-based systems centralized on glutathione/glutaredoxin and thioredoxin function as the primary integrators of redox signaling as they directly and reversibly modulate the oxidation state of protein cysteine residues that encode redox-sensitive switches in the enzymes, ion channels, and transcription factors and classical antioxidant enzymes such as superoxide dismutase and catalase are primarily removes the reactive oxygen species like superoxide and hydrogen peroxide, thereby shaping the overall oxidant milieu. Consequently, the current cardiac redox literature emphasizes that glutathione- and thioredoxin-dependent thiol exchange reactions occupy a more central position in converging and decoding redox cues into functional responses such as cardiomyocyte growth, survival, and adaptation to stress [11].

Despite robust preclinical evidence, clinical trials targeting oxidative stress through antioxidant supplementation have largely failed to demonstrate meaningful benefits in heart failure outcomes. Interventions using vitamin E, vitamin C, and other antioxidant compounds have not consistently improved clinical endpoints, suggesting that nonspecific antioxidant therapy alone is insufficient to counteract the complex and compartmentalized redox disturbances present in heart failure [23].

**Figure 2:**
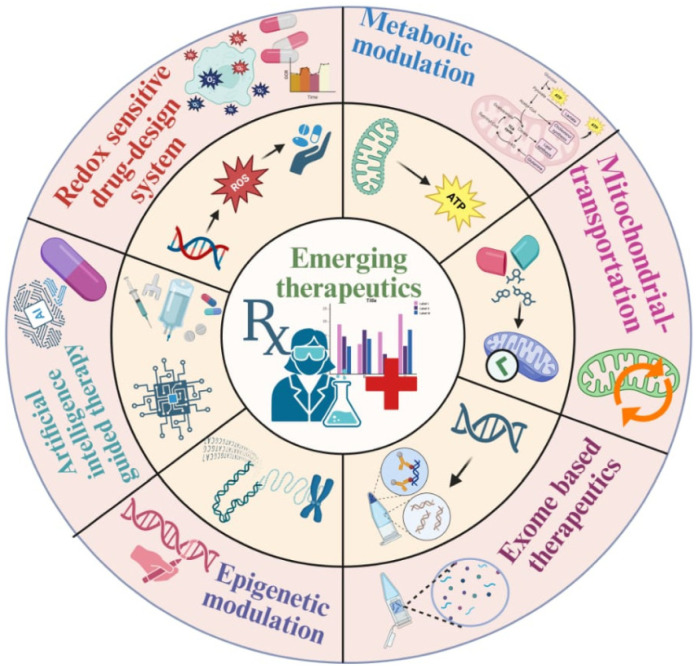
Mechanistically targeted therapeutic strategies addressing redox–genetic dysregulation in heart failure.

Redox signaling interacts closely with genetic susceptibility to drive heart failure progression, as summarized in Figure 2. However, despite consistent associations between oxidative stress markers and disease severity, clinical studies vary widely in patient selection, biomarker assays, and outcome measures, limiting direct comparisons and complicating causal interpretation. Further research integrating redox biology with genetic and molecular profiling is therefore required to develop targeted, mechanism-based therapies that can more effectively address the heterogeneity of heart failure.

Emerging therapeutic approaches target redox imbalance and mutation-driven myocardial dysfunction. These include redox-sensitive drug delivery, metabolic and mitochondrial-directed therapies, exosome-based molecular transfer, and epigenetic modulation to correct oxidative-stress-induced gene dysregulation. Integration of artificial-intelligence-guided precision medicine supports individualized therapeutic strategies aimed at restoring myocardial energetics and redox homeostasis.

### 2.4. Reductive Stress and Cardiac Dysfunction in Patients with HF

Reductive stress is characterized by increased reducing equivalents like inclined GSH/GSSG ratios, NADPH/NADP+, and NADH/NAD+, which disrupts redox homeostasis and contributes to dysfunction of the heart, which is an independent mechanism of oxidative stress [27]. It stimulates protein misfolding, small heat shock protein aggregation (e.g., mutant αB-crystallin R120G), and proteotoxic cardiomyopathy by inhibiting the formation of disulfide bonds and proteasomal/autophagic clearance. This will also lead to maladaptive remodeling, including hypertrophic cardiomyopathy (HCM), diastolic dysfunction, and fibrosis.

Reductive stress often coexists with oxidative stress in heart failure scenarios, which is triggered as a compensatory response (e.g., antioxidant upregulation) that enters a pathological form, resulting in disease progression, exacerbating mitochondrial dysfunction and ER stress in disease states like desmin-related myopathy and ischemia–reperfusion injury. In protein aggregation cardiomyopathies, initial oxidant stress triggers reductive countermeasures, but sustained high GSH perpetuates aggregates and maladaptive changes. Therapeutic modulation, such as Nrf2 inhibition or exercise, can restore balance and mitigate progression [28].

## 3. Genetic Mutations and Heart Failure

### 3.1. Overview of Mutations Linked to Cardiomyopathy and HF

**Figure 3:**
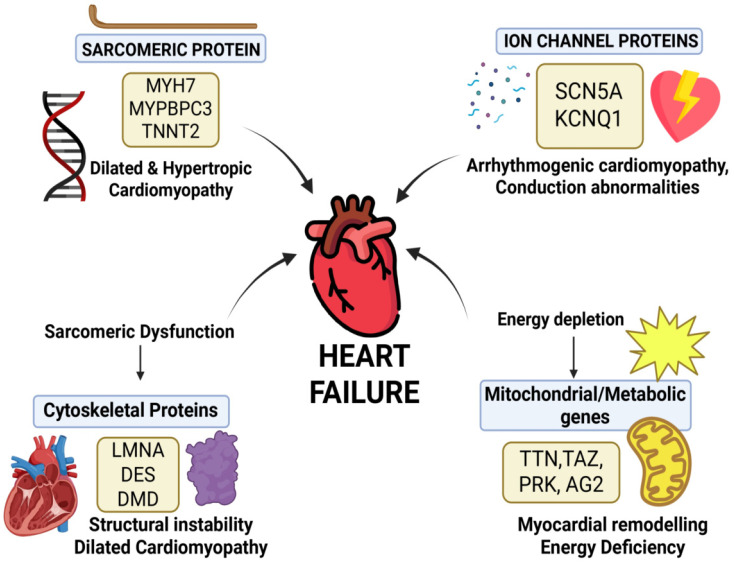
Genetic pathways converging on heart failure.

A wide array of inherited and sporadic mutations contribute to heart failure (HF) syndromes. Familial cardiomyopathies are often linked to rare monogenic variants, while common variants associated with non-familial HF serve as key risk markers [29]. Figure 3 illustrates major gene categories involved in inherited cardiomyopathies and HF, including sarcomeric, ion channel, and cytoskeletal proteins.

Mutations affecting force generation and propagation include defects in sarcomeric proteins (e.g., myosin, actin, troponin, titin) and cytoskeletal components (e.g., desmin, dystrophin). *MYH7*(myosin heavy chain 7) and *TNNT2* (troponin T) mutations are major causes of hypertrophic cardiomyopathy (HCM) and dilated cardiomyopathy (DCM) [30,31,32,33,34,35]. *TTN*-truncating variants are found in up to 25% of DCM cases [36], while *LMNA* (lamin A/C) mutations cause DCM with conduction defects [33]. Beyond structural and electrical roles, mutations in transcriptional and post-translational regulators, such as *RBM20* and *RBM24*, contribute to HF by disrupting RNA splicing, leading to dysregulation of key cardiac genes [34,35]. Filamin C, encoded by the *FLNC* gene, is a critical actin-binding protein expressed in striated muscle that maintains sarcomere integrity and Z-disk stability. Pathogenic variants in *FLNC* are increasingly recognized in restrictive cardiomyopathy and left ventricular non-compaction cardiomyopathy, often expressed in high arrhythmogenic risk and progression to heart failure [37].

### 3.2. Sarcomeric and Cytoskeletal Protein Mutations

Mutations in sarcomeric proteins, particularly *MYH7*, are frequently implicated in cardiomyopathies [38]. *MYH7* variants such as R403Q and R719W enhance ATPase activity, causing hypercontractility and hypertrophy [39,40], whereas mutations like S532P and F764L reduce contractile force, leading to DCM [41]. A 25 mer deletion in MYBPC3 is common in Indian and South Asian populations, emphasizing the importance of regional genetic screening. Troponin mutations also play a crucial role in calcium regulation and cardiomyopathy. *TNNT2* variants increase myofilament calcium sensitivity [42], leading to diastolic dysfunction and arrhythmias [43]. TNNI3 (troponin I) and TNNC1 (troponin C) mutations exhibit similar effects [44], while mutations in TPM1 (tropomyosin) and ACTC1 (actin) contribute to DCM [45] *TTN* truncating variants, which lead to haploinsufficiency, are linked to ~15% of ambulatory and ~25% of end-stage DCM cases [46]. Cytoskeletal protein mutations further exacerbate HF. *DES* (desmin) mutations destabilize the sarcomere and disrupt mitochondrial function [47,48], while dystrophin (*DMD*) mutations, associated with Duchenne muscular dystrophy, result in progressive DCM [49].

### 3.3. Mutations Impacting Redox Homeostasis

Evidence from experimental studies, supported by selected human genetic observations, suggests that several cardiomyopathy-associated mutations directly or indirectly impair redox homeostasis, thereby amplifying oxidative and reductive stress pathways in heart failure. Building on the genetic background outlined in Section 3.1 and Section 3.2, this section focuses specifically on the redox-related consequences of these mutations rather than reiterating primary gene functions.

#### 3.3.1. Structural and Cytoskeletal Mutations Disrupting Redox Balance

Mutations affecting structural and cytoskeletal proteins play a central role in redox dysregulation by altering mechanotransduction, mitochondrial function, and antioxidant defense. *DES* (desmin) mutations impair interactions with antioxidant enzymes such as glutathione peroxidase, resulting in increased oxidative stress, mitochondrial dysfunction, and sarcomeric instability [50].

Beyond its structural role, titin (*TTN*) functions as a redox-sensitive mechanotransducer linking biomechanical stress to intracellular signaling. Pathogenic *TTN* variants, particularly within the elastic I-band region, disrupt stretch-dependent redox signaling and promote excessive mitochondrial reactive oxygen species (ROS) generation [51,52,53]. These alterations predispose cardiomyocytes to maladaptive redox amplification under mechanical load, contributing to progressive dilated cardiomyopathy.

Similarly, *LMNA* (lamin A/C) mutations compromise nuclear integrity and redox regulation, leading to impaired antioxidant responses, increased oxidative stress, and accumulation of DNA damage and lipid peroxidation products [54,55]. Collectively, mutations in these structural proteins establish a permissive substrate for chronic redox imbalance and myocardial remodeling.

#### 3.3.2. Ion Channel and Chaperone Mutations Amplifying Oxidative Stress

Ion channel and protein quality control systems also contribute to redox homeostasis in the myocardium. Mutations in *SCN5A*, particularly the 1795insD variant, have been shown in experimental models to elevate myocardial ROS levels and exacerbate ventricular dysfunction, linking electrical instability with oxidative stress pathways [56,57].

Chaperone proteins with intrinsic antioxidant functions are similarly affected. *CRYAB* (αB-crystallin) normally protects cardiomyocytes from oxidative injury through its chaperone and redox-buffering activity. However, pathogenic *CRYAB* mutations such as R120G impair this protective capacity, leading to increased ROS accumulation, enhanced cardiomyocyte apoptosis, and accelerated heart failure progression [58,59]. These findings highlight how disruption of protein homeostasis can amplify oxidative injury in genetically susceptible hearts.

#### 3.3.3. Mitochondrial Genetic Alterations and ROS Overproduction

Mitochondrial genetic defects represent a direct source of redox imbalance in heart failure. Pathogenic mitochondrial DNA (mtDNA) mutations, including the m.3243A>G tRNA mutation, impair oxidative phosphorylation, reduce ATP generation, and markedly increase mitochondrial ROS production, contributing to severe cardiomyopathic phenotypes [60].

In addition, the age-related accumulation of somatic mtDNA mutations may further exacerbate oxidative stress and increase susceptibility to heart failure, particularly in the presence of nuclear gene mutations affecting redox control. These mitochondrial alterations reinforce a vicious cycle of energetic failure and oxidative injury within cardiomyocytes.

#### 3.3.4. Integrative Perspective and Translational Considerations

Collectively, mutations in structural, electrical, chaperone, and mitochondrial genes converge on redox dysregulation through excessive ROS generation, impaired antioxidant defenses, and mitochondrial dysfunction, thereby accelerating heart failure progression (Figure 3). While experimental models provide strong mechanistic support for these interactions, human genotype–phenotype correlations remain heterogeneous, and the contributions of modifier genes and environmental factors are often difficult to disentangle.

Mutations in sarcomeric genes (*MYH7*, *MYBPC3*, *TNNT2*) impair force generation and drive hypertrophic or dilated cardiomyopathy. Ion channel variants (*SCN5A*, *KCNQ1*) cause electrical instability and arrhythmogenic phenotypes. Cytoskeletal defects (*LMNA*, *DES*, *DMD*) disrupt structural integrity and mechanotransduction. Alterations in mitochondrial and metabolic genes (e.g., *TTN*, *TAZ*, *PRK*, *AG2*) lead to energy deficiency, adverse remodeling, and progression to heart failure.

A synthesis of key genes implicated in heart failure, their associated redox mechanisms, cardiac phenotypes, and potential therapeutic relevance is provided in Table 1, serving as an integrative framework for the redox–genetic axis.

## 4. Interplay of Redox Signaling and Mutations

Genetic mutations in sarcomeric, cytoskeletal, and mitochondrial genes predispose to cardiomyopathies (hypertrophic cardiomyopathy, dilated cardiomyopathy, restrictive cardiomyopathy, arrhythmogenic right ventricular cardiomyopathy), which leads to progression of heart failure by maladaptive remodeling [69].

Controlled ROS normally fine-tunes excitation–contraction coupling, hypertrophy, and adaptation. However, mutation-induced energy inefficiency, dysregulation of Ca^2+^, and mechanosensory defects eventually intensify the pathological ROS from Nox and mitochondria, which creates vicious redox–genetic feedback loops that converge on fibrosis, arrhythmias, and pump failure.

### 4.1. Synergistic Effects on Cardiac Dysfunction

In parallel, ROS activate redox-sensitive kinases, such as Ca^2+^/calmodulin-dependent protein kinase II (CaMKII) and protein kinase A, phosphorylate ryanodine receptors, increase sarcoplasmic reticulum calcium leak, and enhance arrhythmic susceptibility [61,70].

Beyond calcium dysregulation, redox-driven activation of profibrotic signaling cascades, including transforming growth factor-β (TGF-β) and calcineurin–NFAT pathways, promotes interstitial fibrosis, cardiomyocyte hypertrophy, and adverse ventricular remodeling [62,64]. Mitochondrial oxidative stress further amplifies energetic failure and apoptotic signaling, reinforcing a feed-forward cycle of structural deterioration and functional decline. Collectively, these interactions integrate diverse genetic perturbations into common heart failure phenotypes. “Importantly, many proposed synergistic mechanisms are derived from reductionist experimental models and may not fully capture the biological complexity, temporal evolution, and compensatory adaptations characteristic of human heart failure.”

Mechanistic insights from preclinical models demonstrate that genetic susceptibility and redox imbalance synergistically accelerate cardiac dysfunction, promoting the pathways that drive maladaptive remodeling and heart failure progression. Rather than acting independently, mutation-driven vulnerabilities such as sarcomeric defects in *MYH7,* MYBPC3, *TNNT2*, and *FLNC* increase ATP demand and ADP: ATP ratios and impair the efficiency of the electron transport chain and mitochondrial ROS elevation even before overt hypertrophy progresses.

This energetic crisis sensitizes cardiomyocytes to oxidative stress, where excessive ROS from NOX2 and mitochondria modifies key proteins that impair myofilament calcium responsiveness and decrease the contractility by promoting S-glutathionylation of troponin I (TnI) and myosin-binding protein C (MYBPC3) leading to diastolic dysfunction [71].

#### 4.1.1. Calcium Dysregulation

ROS disrupts calcium handling through oxidation of ryanodine receptors (RyR2) via NOX2/CaMKII complexes, causing sarcoplasmic reticulum (SR) Ca^2+^ leaks, delayed after depolarizations, and arrhythmic risk [72]. Mutant myofilaments display hypersensitive or hyposensitive Ca^2+^ responses, amplified by TRPC3-NOX2-mediated stretch-activated local Ca^2+^/ROS signaling during hypertrophy. Redox-sensitive kinases like CaMKII and protein kinase A are activated to further phosphorylates\RyR2, exacerbating SR leak and contractile inefficiency.

#### 4.1.2. Contractile and Structural Defects

Oxidative stress promotes degradation of contractile proteins like MYBPC3 [73] and TnI [74], while filamin C (*FLNC*) mutations destabilize Z-disks, accelerating sarcomere disassembly under ROS burden. Elevated ADP: ATP ratios from sarcomeric inefficiency trigger chronic ATP depletion, reducing force generation and fostering a feed-forward cycle of dysfunction.

#### 4.1.3. Profibrotic Remodeling

Redox-driven activation of TGF-β, calcineurin-NFAT, and NF-κB pathways promotes fibroblast activation, cardiomyocyte hypertrophy. Mutation disrupts normal biophysical properties of sarcomere and causes mechanical and calcium-induced biochemical signals that activate gene transcription, including elevated TGF-β expression. TGF-β stimulates pro-fibrotic molecule expression and proliferation of fibroblasts, which might promote the progression of diastolic dysfunction, which is a clinical hallmark of hypertrophic cardiomyopathy [75]. High levels of ROS trigger the structural modifications of the sarcomere, which impact pump function and progresses the pathogenesis of heart failure [76].

#### 4.1.4. Mitochondrial Amplification

Mitochondrial DNA variants and sarcomeric mutations impair OXPHOS complexes I/II, causing cristae remodeling, mPTP opening, energetic failure, and apoptosis, with human end-stage HF hearts showing elevated ROS, downregulated Nrf2, and mutation-specific redox signatures like increased ADP in MYBPC3 HCM. While reductionist models illuminate these synergies, they may not fully capture human disease complexity, temporal adaptations, or compensatory mechanisms, underscoring the need for translational validation.

### 4.2. Compensatory Mechanisms and Adaptations

Preclinical models, complemented by limited human observations, suggest that certain redox-responsive pathways function as adaptive or compensatory mechanisms in the setting of genetically mediated cardiac stress. Activation of nuclear factor erythroid 2–related factor 2 (Nrf2) signaling has been shown to upregulate antioxidant enzymes, including heme oxygenase-1 (HO-1), thereby mitigating oxidative injury and preserving cardiomyocyte viability [63].

Experimental studies further indicate that enhanced antioxidant responses can partially offset redox imbalance and mechanical stress, resulting in improved cardiac function in selected genetic cardiomyopathy models [65,66,77]. Consistent with these findings, analyses of human end-stage dilated cardiomyopathy hearts demonstrate differential upregulation of antioxidant transcripts associated with more favorable ventricular geometry and function. Overall, while oxidative stress contributes to heart failure progression, context-dependent redox adaptations represent protective mechanisms that could be therapeutically harnessed in genetically susceptible individuals.

## 5. Emerging Therapeutic Horizons

The therapeutic strategies discussed in this section largely reflect preclinical or early translational evidence, with varying degrees of clinical validation in patients with heart failure. Advances in molecular cardiology and translational research have identified several approaches aimed at modulating the redox–genetic axis, offering potential complementary benefits alongside conventional pharmacological and gene-based therapies. An overview of these emerging strategies and their relationship to redox–genetic mechanisms in heart failure is provided.

### 5.1. Redox-Sensitive Drug Delivery Systems

Redox-sensitive drug delivery systems represent a precision-oriented approach by exploiting the oxidative microenvironment of diseased myocardium. These nanocarriers remain stable under physiological conditions but undergo activation in oxidative settings through reactive oxygen species (ROS)-responsive linkers, enabling site-specific release of antioxidants or genetic modulators. Polymeric nanoparticles and lipid-based vesicles have demonstrated enhanced myocardial targeting and reduced systemic toxicity in preclinical cardiovascular models. However, clinical translation remains limited, with challenges related to long-term safety, scalability, and regulatory approval [67,68,78,79].

### 5.2. Metabolic Modulation and Redox Balance

Metabolic remodeling in the failing myocardium contributes directly to mitochondrial dysfunction and redox imbalance. Pharmacologic metabolic modulators such as trimetazidine and perhexiline promote a shift toward glucose oxidation, improving energetic efficiency and indirectly reducing mitochondrial ROS generation. Selected clinical studies have reported modest improvements in functional capacity and myocardial efficiency; however, heterogeneity in patient populations and outcomes highlights the need for redox- and genotype-informed stratification to optimize therapeutic benefit [80,81,82,83].

### 5.3. Mitochondrial-Targeted Strategies

Mitochondrial dysfunction is a central driver of heart failure progression through impaired ATP production, altered calcium handling, and excessive ROS generation. Experimental strategies such as mitochondrial transplantation aim to restore bioenergetic capacity and redox balance in injured myocardium. While preclinical studies demonstrate improvements in mitochondrial function and reductions in oxidative injury, clinical evidence remains limited to early-phase investigations, and the feasibility of widespread clinical application requires further validation [84,85,86].

### 5.4. Exosome-Based Redox Modulation

Exosome-based approaches are included here for their potential to modulate redox-sensitive signaling pathways and gene regulatory networks through the delivery of antioxidant proteins and regulatory microRNAs. Preclinical studies suggest beneficial effects on oxidative stress, inflammation, and cardiomyocyte survival. Nevertheless, these strategies remain largely experimental, with unresolved challenges related to standardization, dosing, targeting specificity, and clinical scalability limiting their current translational relevance [87].

### 5.5. Epigenetic Modulation of Redox-Responsive Genes

Epigenetic regulation represents a critical interface between genetic susceptibility and redox imbalance in heart failure, as oxidative and reductive stress states directly influence chromatin structure and transcriptional control. Epigenetic mechanisms—including DNA methylation, histone acetylation/deacetylation, and non-coding RNA regulation—govern long-term expression of redox-sensitive genes involved in antioxidant defense, mitochondrial function, inflammation, and myocardial remodeling.

Experimental studies demonstrate that oxidative stress alters the activity of epigenetic enzymes such as DNA methyltransferases (DNMTs) and histone deacetylases (HDACs), reinforcing maladaptive transcriptional programs. Conversely, excessive reductive stress may suppress adaptive redox-responsive gene expression. While preclinical models suggest that HDAC and DNMT inhibition can attenuate pathological remodeling and oxidative injury, clinical translation remains limited due to a lack of cardiac specificity, off-target effects, and inter-individual epigenetic heterogeneity. At present, epigenetic therapies should be viewed as conceptually promising but still experimental components of a broader redox–genetic precision framework [88,89,90].

### 5.6. Integrative and Supportive Computational Approaches

Artificial-intelligence-based approaches are relevant to the redox–genetic axis primarily as integrative and supportive tools rather than direct therapeutic modalities. By combining genomic data, redox biomarkers, and clinical parameters, AI-driven models may assist in patient stratification, risk prediction, and optimization of therapeutic decision-making. However, their current role remains investigational and dependent on robust validation, standardized datasets, and clinical interpretability [91].

### 5.7. Translational Considerations

Overall, despite promising preclinical efficacy, many redox-targeted and gene-based therapies face substantial translational barriers, including limited target specificity, off-target effects, safety concerns, and inconsistent results in early clinical trials. Future progress will depend on improved mechanistic integration, rigorous human validation, and precision strategies that align redox modulation with underlying genetic susceptibility.

## 6. Conclusions

The evidence summarized in this review integrates robust experimental findings with available human data, while acknowledging that the clinical translation of many redox–genetic mechanisms remains incomplete and requires further validation. Collectively, the findings highlight a multifaceted and bidirectional relationship in which redox dysregulation amplifies the pathological effects of genetic variants linked to heart failure, while genetic mutations themselves perturb redox homeostasis. This self-reinforcing cycle accelerates disease progression and complicates therapeutic intervention.

Identification of redox-sensitive genetic variations may help define individuals who are more susceptible to oxidative-stress-driven heart failure. In this context, redox biomarkers hold promise for improving early diagnosis, risk stratification, and disease monitoring. Emerging gene-specific and redox-targeted therapeutic strategies show encouraging mechanistic potential, although their clinical utility remains to be established. Looking forward, meaningful progress in this field will require rigorously designed human studies, standardized redox biomarker assessments, and patient stratification approaches that integrate genetic susceptibility with redox phenotyping. Such efforts may ultimately enable precision medicine strategies that support earlier diagnosis, refined risk assessment, and more targeted therapies for patients with heart failure.

## Figures and Tables

**Table 1:** Redox- and Nrf2-sensitive genes associated with cardiomyopathy and heart failure, highlighting oxidative mechanisms, myocardial phenotypes, and therapeutic relevance of targeted antioxidant modulation.

Gene/ Pathway	Primary Redox Mechanism	Dominant Cardiac Phenotype	Key Pathophysiological Consequence	Potential Therapeutic Relevance	Reference
***TTN***	Increased mitochondrial ROS; impaired mechanosensing and proteostasis	Dilated cardiomyopathy (DCM), HF	Sarcomeric instability, energetic inefficiency	Metabolic modulation; redox-sensitive proteostasis targeting	[29,41,46,51,52,53,61,62,63]
***LMNA***	Oxidative DNA damage; nuclear redox stress	DCM with conduction disease	Nuclear envelope instability; premature senescence	Antioxidant strategies; DNA damage response modulation	[30,33,50,54,55]
***MYH7***	ROS-sensitive sarcomeric dysfunction	Hypertrophic cardiomyopathy (HCM), HF	Impaired force generation; maladaptive hypertrophy	Sarcomere modulators; redox signaling attenuation	[30,31,32,38,39,40,41]
***PLN***	Oxidative and post-translational modification	Systolic HF	SERCA2a inhibition; Ca^2+^ overload	SERCA2a activation; Ca^2+^ redox tuning	[32,48,49]
***RYR2***	Oxidation of regulatory cysteine residues	Arrhythmia-associated HF	Diastolic Ca^2+^ leak; electrical instability	RyR2 stabilizers; targeted antioxidant delivery	[31,47,51,59]
***ATP2A2 (SERCA2a)***	ROS-mediated transcriptional downregulation	HF with impaired relaxation	Reduced SR Ca^2+^ reuptake	Gene therapy; metabolic and redox modulation	[32,48,49]
***CRYAB***	Reductive/oxidative imbalance; impaired chaperone activity	Proteotoxic cardiomyopathy	Protein aggregation; myocyte dysfunction	Proteostasis enhancement; redox balancing strategies	[52,53,58,64]
***NFE2L2 (Nrf2)***	Dysregulated antioxidant signaling	HFpEF; metabolic HF	Maladaptive or insufficient redox adaptation	Precision Nrf2 pathway modulation	[11,65,66,67,68]
***SCN5A***	ROS-induced sodium channel dysfunction	Arrhythmogenic cardiomyopathy	Electrical conduction abnormalities	Redox-sensitive ion channel modulation	[36,56,57,67]
***NOX2/NOX4***	Excess ROS generation	HF progression; myocardial fibrosis	Oxidative injury; inflammatory signaling	Isoform-specific NADPH oxidase inhibition	[58,60]

DCM: dilated cardiomyopathy; HCM: hypertrophic cardiomyopathy, HF: heart failure; HFpEF: heart failure with preserved ejection fraction; ROS: reactive oxygen species; SR: sarcoplasmic reticulum; Ca^2+^: calcium ion; SERCA2a: sarco/endoplasmic reticulum Ca^2+^-ATPase 2a; *NFE2L2*: ryanodine receptor 2; Nrf2: nuclear factor erythroid 2–related factor 2; NOX: NADPH oxidase.

## Data Availability

No new data were created or analyzed in this study. Data sharing does not apply to this article.
